# Recent beak evolution in North American starlings after invasion

**DOI:** 10.1038/s41598-023-49623-y

**Published:** 2024-01-02

**Authors:** Julia M. Zichello, Shelagh T. DeLiberto, Paul Holmes, Agnieszka A. Pierwola, Scott J. Werner

**Affiliations:** 1 Hunter College, City University of New York, New York, NY USA; 2 Division of Anthropology, American Museum of Natural History, New York, NY USA; 3 United States Department of Agriculture, Animal and Plant Health Inspection Service, Wildlife Services, National Wildlife Research Center, Fort Collins, CO USA; 4 Animal and Plant Health Agency, Shrewsbury Veterinary Investigation Centre, Shrewsbury, SY1 4HD UK; 5 Division of Invertebrate Zoology, American Museum of Natural History, New York, NY USA

**Keywords:** Evolutionary ecology, Invasive species

## Abstract

European starlings are one of the most abundant and problematic avian invaders in the world. From their native range across Eurasia and North Africa, they have been introduced to every continent except Antarctica. In 160 years, starlings have expanded into different environments throughout the world, making them a powerful model for understanding rapid evolutionary change and adaptive plasticity. Here, we investigate their spatiotemporal morphological variation in North America and the native range. Our dataset includes 1217 specimens; a combination of historical museum skins and modern birds. Beak length in the native range has remained unchanged during the past 206 years, but we find beak length in North American birds is now 8% longer than birds from the native range. We discuss potential drivers of this pattern including dietary adaptation or climatic pressures. Additionally, body size in North American starlings is smaller than those from the native range, which suggests a role for selection or founder effect. Taken together, our results indicate rapid recent evolutionary change in starling morphology coincident with invasion into novel environments.

## Introduction

European starlings (*Sturnus vulgaris*) were introduced into North America in 1890 and 1891 from their native range in Europe. From a small founder population of approximately 100 birds, their population expanded to ~ 200 million within a century but has recently been estimated between 85.9 and 93.3 million birds^1–3^. By 1942, starlings had spread to the West Coast of the United States and now inhabit all U.S. states, most of Mexico, and parts of Southern Canada^4^. They were also introduced to New Zealand, Australia, South Africa, and Argentina (between 1856 and 1987)^5–7^. A recent study estimated that European starlings are the second most abundant wild bird in the world, with a global population of 1.3 billion, ranked behind only the house sparrow (*Passer domesticus*) at 1.6 billion^8^.

These multiple successful introductions of European starlings to different parts of the world make this species an exemplar of adaptation to new environments, population size expansion, and dispersal. This multi-continental single-species system can therefore offer important windows into the ecological, genetic, behavioral, and physiological variables which enable avian invasions—and, more broadly, drive evolutionary change over short time scales. Importantly, European starlings in the U.S. pose substantial ecological and economic problems by consuming agricultural crops, spreading diseases to livestock and consuming their food, colliding with aircraft, and competing with native birds such as Sapsuckers (*Sphyrapicus* sp)^9–16^. Therefore, studying this species from a comparative population perspective serves two key purposes: to better understand evolutionary change over short time scales and to illuminate aspects of their invasion history and population dynamics which can inform management efforts and conservation of native species.

Consistent with their invasion history, even a modest sampling of birds from the United Kingdom shows higher mitochondrial haplotype diversity (*h* = 0.972) and nucleotide diversity (π = 0.7%), than the invasive populations in North America (*h* = 0.876, π = 0.5%), Australia (*h* = 0.703, π = 0.5%), or South Africa (*h* = 0.779, π = 0.5%)^17–19^. Although, due to a lack of comprehensive genetic data from starlings across the entirety of their native range, the full extent of their native genetic diversity is not well characterized. Therefore, comparisons of genetic diversity between native and invasive ranges are currently provisional. Relatedly, the source population for many of these introductions is historically documented as Britain, but this has not been confirmed with genetic data^7^. Comparisons between invasive ranges show that North America (NA) harbors higher mitochondrial haplotype diversity than Australia or South Africa, which may be due to rapid expansion in NA facilitated by climate matching with the native range. Yet, the effective population size, estimated from nuclear markers, is slightly larger in Australia than in North America, possibly due to differences in propagule pressure between invasive ranges^20^.

There are ten recorded starling introductions to North America^7^. According to historical records, the only populations which became established were those from the introductions to New York (1890, 1891) and Oregon (1889, 1892). Those in Oregon persisted only until 1900. Genetic analyses of starlings in the United States have revealed a lack of geographic population structure using allozymes, mitochondria, and nuclear markers^17,20,21^. The apparent lack of geographic population genetic structure across the U.S. today does not refute a single New York origin for modern starlings across the entire continent. This large panmictic North American population is also likely maintained by east–west and sometimes erratic, opportunistic, migratory patterns^22^. This contrasts with Australia where repeated introductions, beginning in 1865 and continuing until 1881, are at least partly evident in two geographically restricted genetic groups today^18,23^.

Clearly, the relatively small founder population size of starlings in North America and subsequent loss of genetic diversity was not an obstacle to their invasion success, a trend which has also been observed in other invasive species^24–28^. The North American starling population experienced a reduction in genetic diversity followed by a rapid expansion, as shown with reduced representation genomic data^20^. This rapid rate of population growth following the starlings’ arrival in NA may have enabled an escape from the deleterious impact of genetic drift and, instead, provided the conditions for adaptive change. Genotype-environment analyses have identified a suite of single-nucleotide polymorphisms which are correlated with temperature and precipitation in both NA and Australian starling populations^20,29^. This suggests that continent-wide climatic variation may have contributed to heterogenous spatial patterns in starlings driven by adaptation, and which may be evident in external morphometric traits. This idea is supported by an earlier study of 168 starlings across NA, where wing length, beak length and other aspects of body composition covaried with latitude^30^. Introduced starlings in New Zealand also exhibit geographic variation in morphometric traits such as bill size and shape^31^. And more broadly, intraspecific clinal variation in bird body size has been shown to be driven by maximum annual summer temperatures across several taxa, although determining the contribution of selection versus plasticity remains a challenge^32,33^.

The unique population history of European starlings in North America, together with results from recent genetic analyses which suggest an environmental signal of selection, provide the framework for our exploration of their morphological variation. Here, three fundamental questions were addressed: (1) Is starling morphology in North America different than that of the population in the native range? (2) Has starling morphology changed since the time of their introduction to North America? (3) Is modern starling morphology spatially structured across the United States? We measured beak length, wing chord and tarsus length in 1217 birds. Historical specimens dating back to 1816 were measured from museum ornithology collections. Measurements from modern NA starlings collected at dairies and feedlots were also included (provided by the United States Department of Agriculture, Animal and Plant Health Inspection Service, Wildlife Services program; USDA-APHIS-Wildlife Services). Measurements were also collected from modern starlings from Wales, UK (provided by APHA Shrewsbury, Veterinary Investigation Centre, Shrewsbury, UK). The primary comparison here is between birds from North America and those from the native range in Eurasia, though we also included specimens from the starling population in New Zealand which provided another independent invasive population as comparison.

## Materials and methods

### Specimens

All specimens included in this study were identified by adult plumage. Data were collected from a total of 743 museum skins spanning 201 years (females = 286, males = 397, sex N/A = 60) with an additional 337 fresh specimens (females = 116, males = 221) supplied by the USDA-APHIS-Wildlife Services, and 137 fresh specimens supplied by APHA Shrewsbury (sex N/A) (Table 1).

**Table 1:** Starling specimens used in this study: historical skins from museum collections and modern birds.

Starling specimens	specimen type	N	Females	Males	Sex N/A	Localities	Dates collected
**North America (NA)**						*US states*	
AMNH—NA	museum historical	123	50	68	5	AK, CT, FL, IL, MA, MD, NJ, NY, PA, RI, WI	1889–2015
DMNH—NA	museum historical	44	18	22	4	CO	1939–2016
FMNH—NA	museum historical	289	128	134	27	IL, MI, WI	1938–2018
USDA—NA	modern	315	111	204	–	AZ, CA, CO, IA, ID, IL, KS, MN, MO, NE, NC, NH, NV, NY, OR, TX, VT, WA, WI	2016, 2017
USDA—NA	modern	22	5	17	–	AZ	2020
**Native range**						*Countries*	
AMNH—native range	museum historical	101	21	73	7	England, Germany, Latvia, Scotland, Sweden	1816–1965
FMNH—native range	museum historical	11	4	7	-	England, Germany	1888–1921
NHM, Tring—native range	museum historical	107	38	57	12	Azerbaijan, England, Germany, India, Iran, Iraq, Latvia	1859–1975
APHA—native range	modern	137	-	-	137	Wales, UK	2021, 2022
**New Zealand**						*Regions*	
MONZ—NZ	museum historical	68	27	36	5	North Island, South Island, Raoul Island, Ocean Island	1927–2017

**Figure 1:**
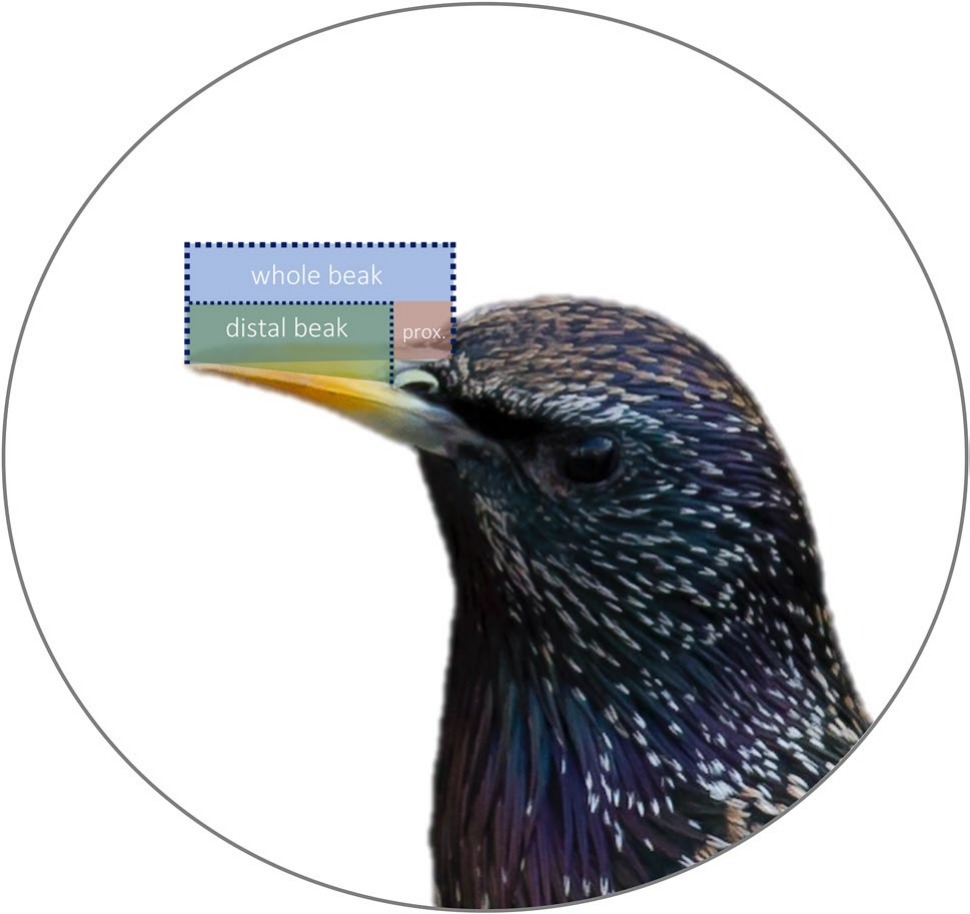
Starling measurements: whole beak, distal beak, proximal beak. Image adapted from Wikimedia Commons, by Pierre Selim.

Measurements taken on all specimens were: whole beak length (or culmen length) originating at the base of the cranium, distal portion of the beak (distance between nares and distal tip of the beak), tarsus length and wing chord length^34,35^ . The measurement for the proximal portion of the beak, from the base of the cranium to the nares, was calculated by subtracting the distal beak measurement from the whole beak measurement (Fig. 1). Digital calipers were used for beak and tarsus measurements and stopped wing rulers were used for wing chord measurements. All digital caliper measurements were taken in millimeters to the nearest 0.01 mm, and wing chord was measured to the nearest 0.5 mm. Digital calipers were zeroed between each measurement. Each measurement was taken three times for each bird, and the average of the three measurements was used.

Museum skins were accessed from the collections of the American Museum of Natural History (AMNH), Field Museum of Chicago (FMNH), Denver Natural History Museum (DMNH), Natural History Museum at Tring (NHM), UK and Te Papa Museum in New Zealand (MONZ). Museum specimens from the native range included birds from England, Germany, Latvia, Azerbaijan, Iran, Iraq, and India. Subspecies distinctions have been assigned to populations from across the native range, though this classification system has not been recently reviewed. The temporal ranges of our datasets from museum specimens are: 159 years (1816–1975) for the native range; 127 years for North America (1890–2017); and 90 years for New Zealand (1927–2017). To compare with the data from museum specimens, 337 modern starlings collected from January-March 2016 and January–February 2017 at animal agriculture facilities within 20 states across the U.S. were also included (Table 1). An additional 22 starlings were collected from a landfill in Arizona in January 2020 (See supplemental material).

Three to fifteen birds were collected from each agricultural facility site, at 2–8 sites per state, and sites were selected ≥ 5 km from nearest sites to maximize the geographic range of sampling. Sample sizes greater than 15 birds were only collected from single sites within three states: Texas, Nevada, and Arizona. United States Department of Agriculture’s Wildlife Services personnel used a handheld GPS unit to record the latitude and longitude of each collection site for subsequent spatial analyses.

The National Wildlife Research Center’s (USDA-APHIS-Wildlife Services) Institutional Animal Care and Use Committee approved the collection and use of starlings for this study (QA-2572, S.J. Werner- Study Director). Samples were utilized for multiple research objectives^20,36^. Live-trapped starlings in the U.S. were euthanized using procedures approved by the American Veterinary Medical Association, and in accordance with ARRIVE guidelines (e.g., C0_2_). All methods were performed in accordance with the relevant guidelines and regulations. These bird collections were authorized by Federal Depredation Permits issued by the U.S. Fish and Wildlife Service and, where necessary, by state scientific collection licenses. Because European starlings are invasive to the U.S., no federal scientific collecting permit was necessary for these collections. Additionally, this collection of starlings likely had no effect on the overabundant populations of starlings often found in association with animal agriculture facilities^2^.

Initial practice measurements were performed with three museum specimens and a sub-sample of five birds^37^. Once accurate replication among observers was achieved, morphometric measurements of 337 starlings were taken by three individuals in July–August 2018. Starlings were then frozen and maintained in chest freezers at − 20 °C. Birds were aged and sexed using plumage characteristics as described by Blasco-Zumeta and Heinze^38^.

Modern starling specimens (N = 137) were also included from the native range in Wales, UK, from salvage birds that died in collisions in 2021 and 2022 (provided by APHA Shrewsbury, Veterinary Investigation Centre, Shrewsbury, UK).

### Ethics declarations

The collection of live starlings for this study was approved by the National Wildlife Research Center’s (USDA-APHIS-Wildlife Services) Institutional Animal Care and Use Committee (QA-2572, S.J. Werner- Study Director). All birds were euthanized using procedures approved by the American Veterinary Medical Association and authorized by Federal Depredation Permits issued by the United States Fish and Wildlife Service and, where necessary, by state scientific collection licenses.

## Data analysis

### Correction for museum skins versus modern specimens

Our analyses included both museum skin specimens and fresh specimens. Shrinkage over time in museum skins has been reported in various passerine bird species, such as house sparrows (*Passer domesticus*), Tennessee warblers (*Leiothlypis peregrina*), and great grey shrikes (*Lanius excubitor*)^39–41^. The precise degree of shrinkage is specific to certain anatomical elements, with wings showing more consistent trends of shrinkage than tarsi or beaks, due to the inclusion of a boney joint in the wing^40^. To address this issue and combine museum and fresh samples into one analysis, correction factors have been derived. These correction factors are ideally meant to be species-specific and are related to nuances in bird body size, morphology, and soft tissue anatomy^40,42^. Without a starling-specific correction factor for museum specimen shrinkage, we used an average for each measurement which is derived from the three aforementioned passerine species. We applied correction factors to all measurements taken from fresh specimens, where we multiplied beak length measurements by 0.969, wing chord by 0.985 and tarsus by 0.975. We then performed all analyses with these correction factors in place.

### Sexual dimorphism

#### ANOVA [location (sex)]

To evaluate the contribution of sexual dimorphism to the variance in each trait, three ANOVA tests were run. One for each location (United States, native range, New Zealand) with sex as an independent qualitative variable, and the five measurements (wing length, tarsus length, whole beak length, proximal beak length, distal beak length) as dependent variables.

#### Change over time

##### Fixed effect regression model

To further parse the relationships between location, sex, time, and each of the five measurements, fixed effect regressions were performed where we controlled for body size.

All 1217 specimens were included here. First, a principal component analysis was run which included all five measurements together. Then, PC1 scores for each individual bird were extracted and used as a fixed effect in the model to control for body size^43^. Time (since 1816, the oldest specimen in our study), location (North America, native, New Zealand) and sex (F, M, N/A) were included as interaction terms, and each measurement was a dependent variable.

$$\left( {{\text{measurement}}} \right) \sim \left( {{\text{PC1}}} \right) + \left( {{\text{time}}} \right)*\left( {{\text{location}}} \right)*\left( {{\text{sex}}} \right)$$

##### Linear regressions

**Figure 2:**
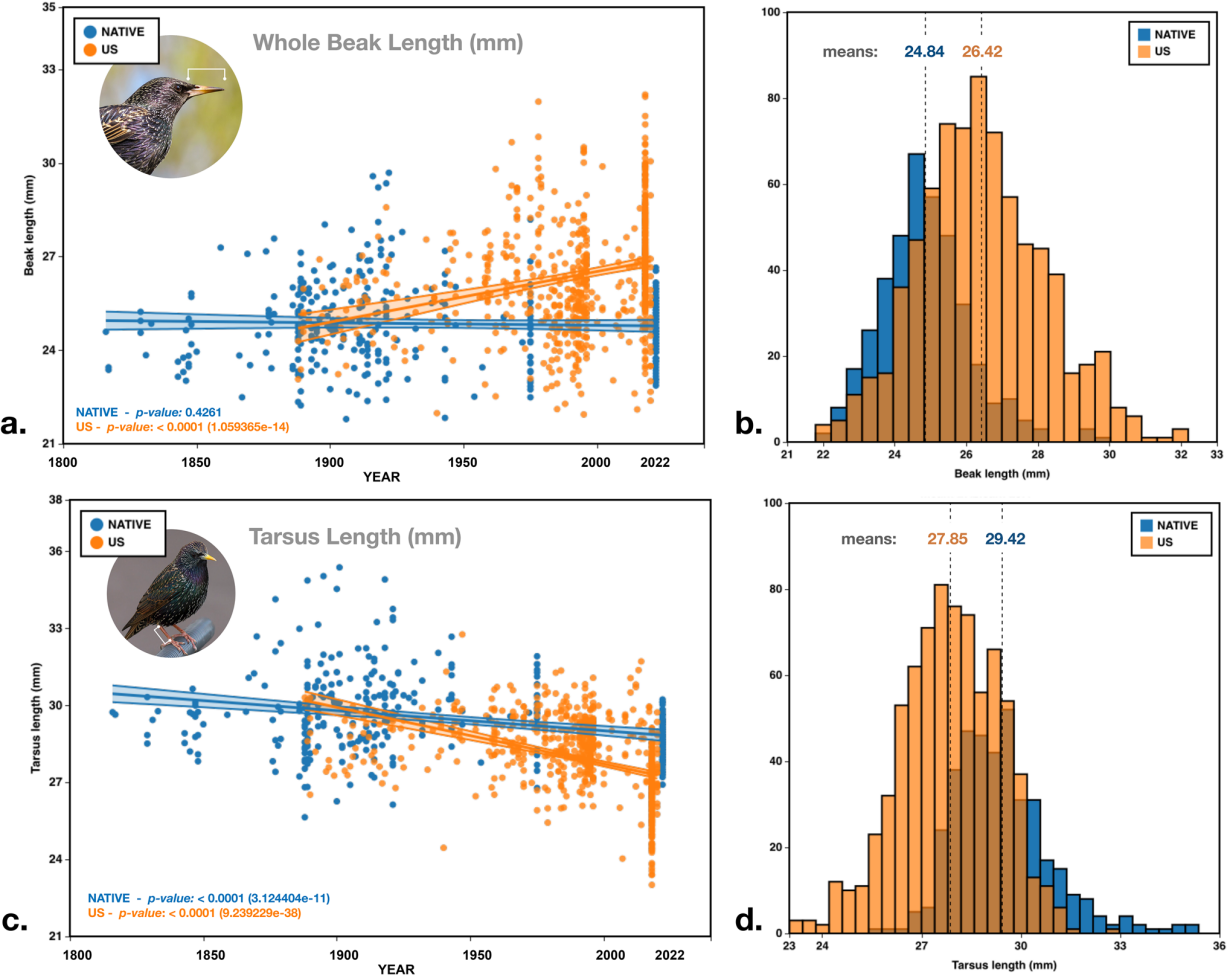
(**a**) Whole beak length (mm) over time, native range 1816–2022 (blue), *p value* = 0.4261; introduced U.S. range 1890–2020 (orange), *p value* < 0.0001 (1.059365e−14). (**b**) Histogram of whole beak length (mm), native range (blue), introduced U.S. range (orange). (**c**) Whole tarsus length (mm) over time, native range 1816–2022 (blue), *p value* < 0.0001 (3.124404e−11); introduced U.S. range 1890–2020 (orange), *p value* < 0.0001 (9.239229e−38). (**d**) Histogram of whole tarsus length (mm), native range (blue), introduced U.S. range (orange).

**Figure 3:**
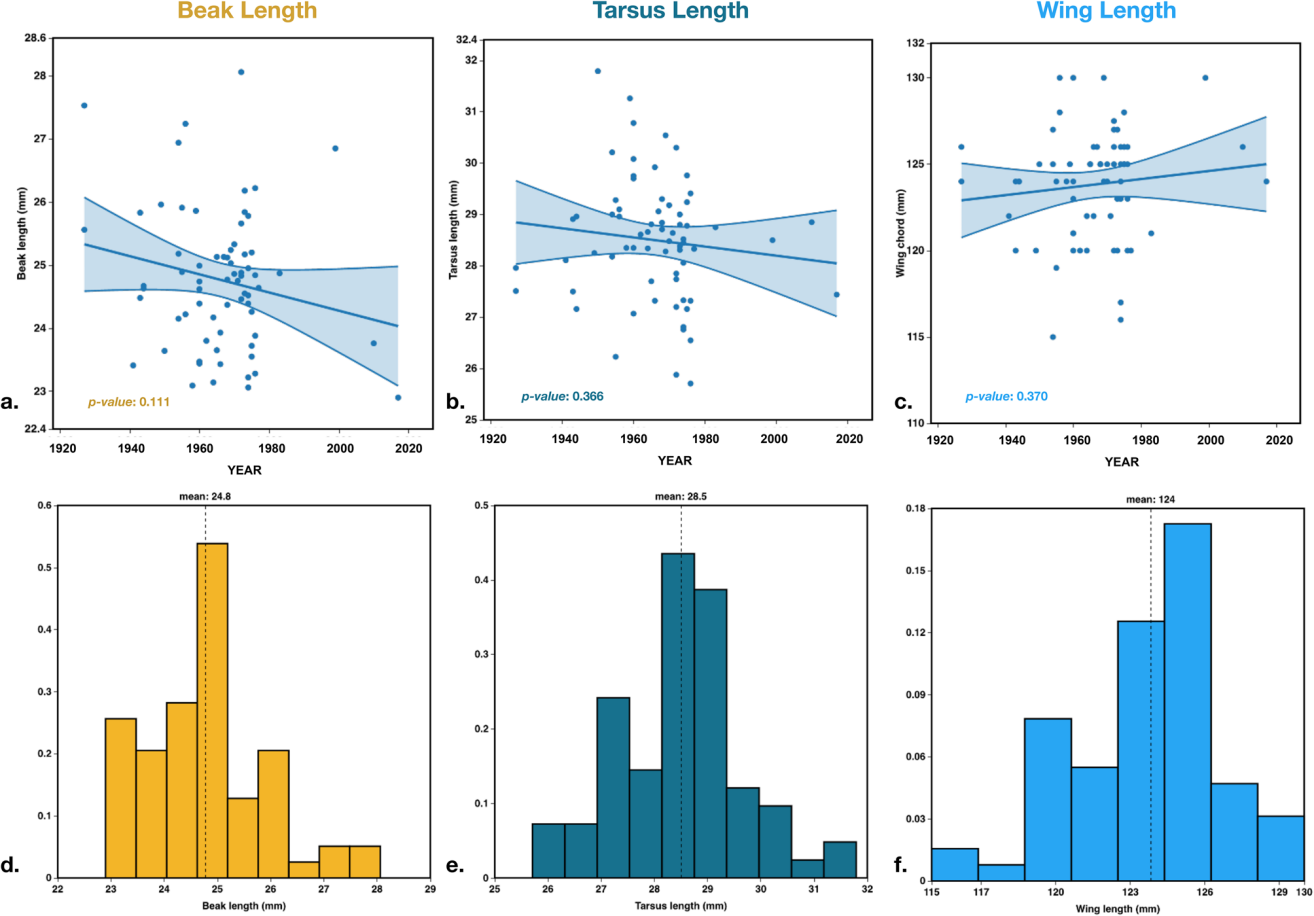
(**a**) New Zealand: whole beak length (mm) over time, 1927–2017, *p value* = 0.111. (**b**) New Zealand: tarsus length (mm) over time, 1927–2017, *p value* = 0.366. (**c**) New Zealand: wing length (mm) over time, 1927–2017, *p value* = 0.370. (**d**) Histogram of beak lengths (mm) from New Zealand. (**e**) Histograms of tarsus lengths (mm) from New Zealand. (**f**) Histograms of wing lengths (mm) from New Zealand.

We also performed linear regressions with time as a continuous variable with each of the five measurements (wing length, tarsus length, whole beak length, proximal beak length, distal beak length) partitioned by different regions: Native range dataset, 206 years (1816–2022) N = 392; North American dataset 130 years (1890–2020) N = 758; and New Zealand 90 years (1927–2017) N = 68 (Figs. 2a,c, 3a–c).

### Comparisons between native range and invasive U.S. population

#### 20 years after introduction to North America

**Figure 4:**
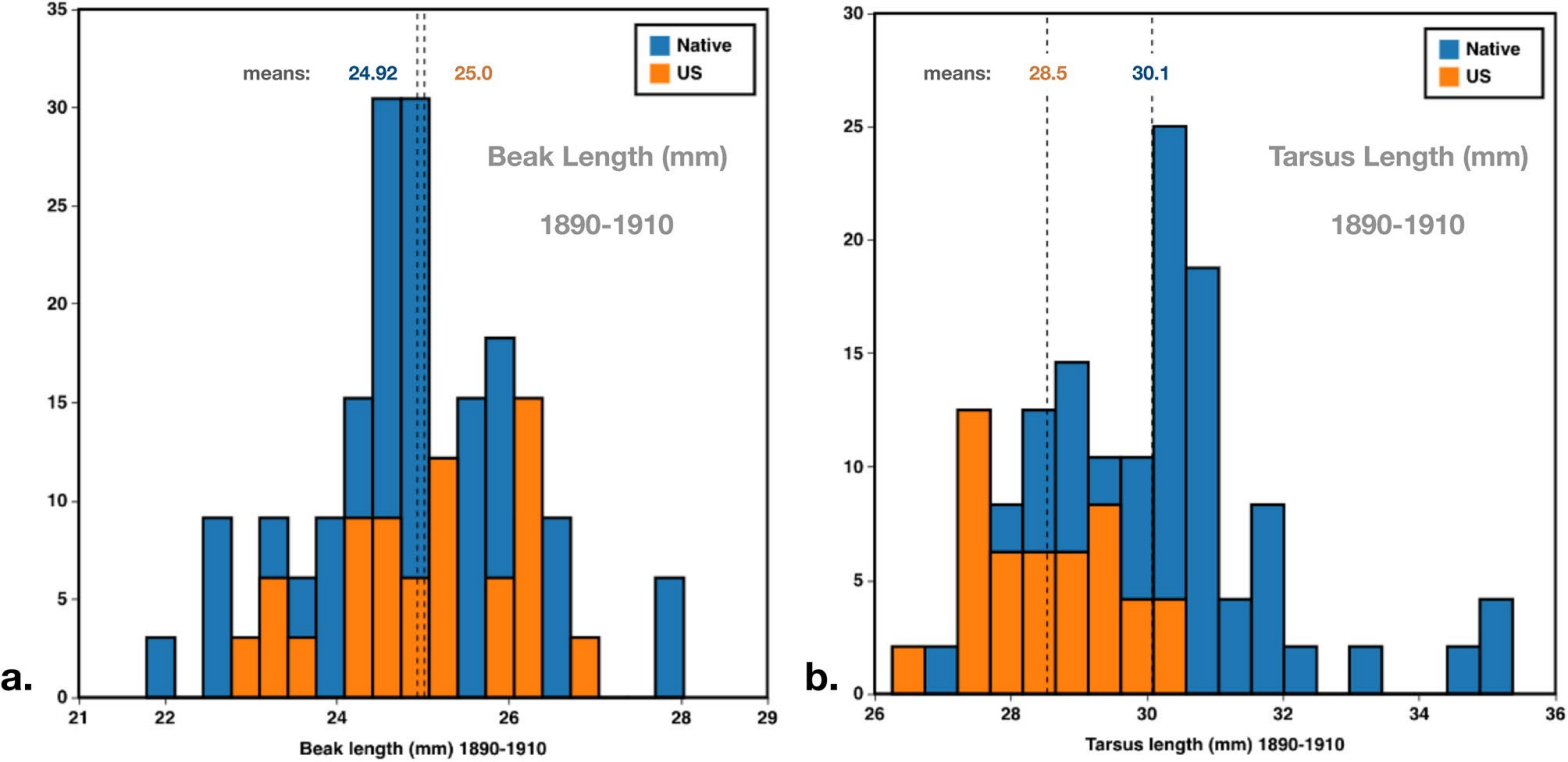
(**a**) Histograms of beak lengths (mm) from 1890 to 1910 only, native range (blue), introduced U.S. range (orange). (**b**). Histograms of tarsus lengths (mm) from 1890 to 1910 only, native range (blue), introduced U.S. range (orange).

To determine if differences between populations can be observed shortly after introduction of starlings to the United States, the North American and native range datasets were subsampled to include the same overlapping 20-year period from 1890 to 1910 (native range, N = 53; North America, N = 24), and two-tailed T-tests were performed to compare differences between population means (Table 2 bottom, Fig. 4).

**Table 2:** Means for all starling measurements in this study organized by region (North America, native range, New Zealand), derived from raw data for museum specimens, and corrected values for fresh specimens (to account for potential shrinkage and enable comparison with data from historical specimens).

Means (raw data)	Beak length (mm)	Tarsus length (mm)	Distal beak Length (mm)	Proximal beak length (mm)	Wing length (mm)
*North America (NA) all*	*26.42*	*27.85*	*18.25*	*8.17*	*123.10*
NA museum skins	25.88	28.65	18.37	7.52	123.55
NA, USDA 2017	27.33	26.68	18.09	9.24	122.63
NA, USDA 2020—Arizona	25.20	27.54	18.06	7.14	120.19
NA 1890–1910	25.02	28.53	18.16	6.85	123.96
NA, USDA 2017, East	27.62	26.95	18.28	9.34	123.34
NA, USDA 2017, Central	27.51	26.94	18.16	9.35	122.70
NA, USDA 2017, West	27.37	26.53	17.94	9.43	122.37
NA, USDA 2017, North	27.55	26.90	18.14	9.41	123.10
NA, USDA 2017, South	27.28	26.46	17.97	9.31	121.62
*Native Range all*	*24.84*	*29.42*	*17.83*	*7.01*	*126.20*
Native range museum skins	24.90	29.86	17.61	7.29	125.24
Native range Europe only	24.65	29.81	17.47	7.18	125.04
Native range Asia only	26.25	30.08	18.37	7.88	126.33
Native range 1890–1910	24.79	30.12	17.68	7.11	125.89
Native range modern UK	24.73	28.62	18.25	6.48	127.97
*New Zealand*	*24.77*	*28.50*	*16.49*	*8.28*	*123.82*

T-Tests (two-tailed)	Beak length	Tarsus length	Distal beak length	Proximal beak length	Wing length
**North America (1890–1910)** Native range (1890–1910)	*0.408*	** < 0.001**	**0.046**	*0.265*	**0.036**
**North America (2016, 2017, 2020)** Native range UK (2021, 2022)	** < 0.001**	** < 0.001**	*0.112*	** < 0.001**	** < 0.001**

P-values from T-tests comparing means between two populations, non-significant values in italics.

Significant values are in bold.

#### Modern starlings from native range compared with modern starlings in the U.S.

Average beak lengths from modern live-caught birds from the UK (2021, 2022) were compared to modern live-caught birds from the U.S. (2017, 2020) only. We generated a random subsample of 137 U.S. birds from those caught on agricultural facilities in 2018, combined with those caught at a non-agricultural facility in 2020. This was compared with 137 live-caught birds from a non-agricultural facility in Wales, UK (2021, 2022). Two tailed T-tests were performed to determine if the differences between means were significant (Table 2 bottom).

### Comparison of modern starlings across the United States (East, Central, West; North, South)

**Figure 5:**
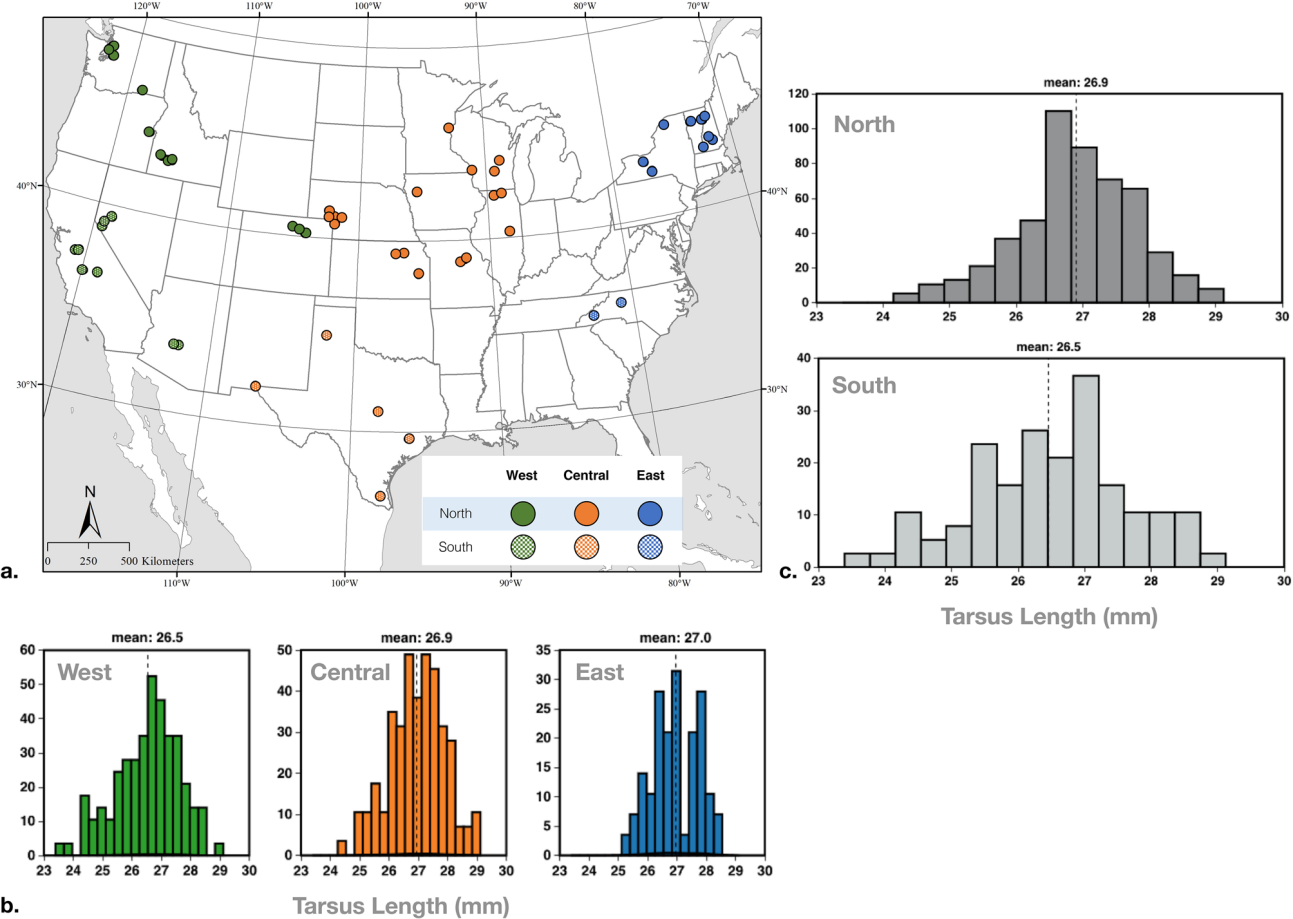
(**a**) Map of USDA fresh specimen localities collected in winter 2017 only, data split by regions: West (green), Central (orange), East (blue); North (solid), South (dotted). Map created with ArcGIS Pro (software) 3.0.2 (version); source data for map features from ESRI ArcGIS Living Atlas (https://livingatlas.arcgis.com/en/home/). (**b**) Histograms of tarsus length (mm) differences between West, Central and East regions of the U.S. (**c**) Histograms of tarsus length (mm) differences between North and South U.S. regions.

Data from modern U.S. starlings were compared between the eastern, central, and western states and between northern and southern states (all from winter 2017, collected by USDA). Specimens were geographically grouped based on the National Oceanic and Atmospheric Administration (NOAA) U.S. Climate Regions Map. ANOVAs were performed to test for differences between east, central and west, and between north and south for all five measurements (Table 4, Fig. 5a).

**East** includes NOAA regions Northeast and Southeast: Vermont (VT), New Hampshire (NH), New York (NY), and North Carolina (NC). **Central** includes NOAA regions Upper Midwest, Ohio Valley, South, Northern Rockies and Plains: Wisconsin (WI), Illinois (IL), Minnesota (MN), Iowa (IA), Missouri (MO), Nebraska (NE), Kansas (KS), and Texas (TX). **West** includes NOAA regions Northwest, West, and Southwest: Idaho (ID), Nevada (NV), Colorado (CO), Arizona (AZ), Washington (WA), Oregon (OR), and California (CA). **North** includes NOAA regions Northeast, Ohio Valley, Upper Midwest, Northern Rockies and Plains, and Northwest: VT, NH, NY, WI, IL, MN, IA, MO, NE, KS, CO, ID, WA, and OR. **South** includes NOAA regions Southeast, South, Southwest, and West: NC, AZ, TX, NV and CA (Fig. 5a).

### Software

All analyses were performed using the XLSTAT Add-in for Microsoft Excel, Version 16.54. Figures were generated with BioVinci 3.0.9, Bioturing Data Visualization Software, 2020.

## Results

### Sexual dimorphism

#### ANOVA [location (sex)]

**US:** ANOVA results for United States with SEX as qualitative variable showed no R^2^ value exceeded 0.08. Sex explained 8% of variance in total beak length and wing length: **beak length** (R^2^ = 0.08, F_SEX_ = 63.27, *p value* =  < 0.0001), **wing length** (R^2^ = 0.08, F_SEX_ = 63.02, *p value* =  < 0.0001). Sex explained 6% of the variance in **distal beak length** (R^2^ = 0.06, F_SEX_ = 43.23, *p value* =  < 0.0001), 1% of the variance in **proximal beak length** (R^2^ = 0.01, F_SEX_ = 8.97, *p value* = 0.003), and was not significant for tarsus length (R^2^ = 0.001, F_SEX_ = 0.99, *p value* = 0.321).

**NATIVE RANGE:** ANOVA results for the native range with SEX as qualitative variable showed no R^2^ value exceeded 0.07. Sex explained 7% of the variance in **wing length** (R^2^ = 0.07, F_SEX_ = 16.25, *p value* =  < 0.0001), 6% of variance in **beak length** (R^2^ = 0.06, F_SEX_ = 15.16, *p value* = 0.0001), 4% of variance in **proximal beak length** (R^2^ = 0.04, F_SEX_ = 10.77, *p value* = 0.001), 2% of **distal beak length** (R^2^ = 0.02, F_SEX_ = 4.02, *p value* = 0.05), and was not significant for tarsus length (R^2^ = 0.02, F_SEX_ = 4.30, *p value* = 0.04).

**NZ:** ANOVA results for New Zealand with SEX as qualitative variable showed only one significant result where sex explained 7% of variance in **wing length** (R^2^ = 0.07, F_SEX_ = 4.94, *p value* = 0.03). All other traits for New Zealand _SEX_ were non-significant: beak length (R^2^ = 0.01, F_SEX_ = 0.53, *p value* = 0.47), proximal beak length (R^2^ = 0.02, F_SEX_ = 1.00, *p value* = 0.32), distal beak length (R^2^ = 0.03, F_SEX_ = 1.74, *p value* = 0.19), tarsus length (R^2^ = 0.05, F_SEX_ = 3.06, *p value* = 0.09). ANOVA summary data in Table 3, LS means in Table 4.

**Table 3:** Results from ANOVA with data separated by location (U.S., native, NZ) and sex within each location, and ANOVA results for U.S. regions only (north–south, east-central-west).

ANOVA	Beak length	Tarsus length	Distal Beak length	Proximal beak length	Wing length
**ALL LOCATIONS**					
US (F + M)					
R^2^	0.08	0.001	0.06	0.01	0.08
F	63.27	0.99	43.23	8.97	63.02
Pr > F	** < 0.001**	*0.321*	** < 0.001**	**0.003**	** < 0.001**
NATIVE (F + M)					
R^2^	0.06	0.02	0.02	0.04	0.07
F	15.16	4.30	4.02	10.77	16.25
Pr > F	**0.0001**	**0.04**	**0.05**	**0.001**	** < 0.0001**
NZ (F + M)					
R^2^	0.01	0.05	0.03	0.02	0.07
F	0.53	3.06	1.74	1.00	4.94
Pr > F	*0.47*	*0.09*	*0.19*	*0.32*	**0.03**
**US REGIONS ONLY**					
NORTH–SOUTH					
R^2^	0.01	0.03	0.005	0.01	0.05
F	3.71	9.46	1.35	2.27	14.26
Pr > F	*0.06*	** < 0.001**	*0.25*	*0.13*	** < 0.001**
EAST–CENTRAL–WEST					
R^2^	0.004	0.04	0.01	0.002	0.01
F	0.56	5.77	1.47	0.33	1.61
Pr > F	*0.57*	** < 0.001**	*0.23*	*0.72*	*0.20*

Significant values in bold, non-significant values in italics.

**Table 4:** Summary statistics from ANOVA with all data separated by location (and sex within each location).

All data (from ANOVA)	LS mean	Standard error	Lower bound	Upper bound
**BEAK LENGTH**				
US (all)	26.11	0.08	25.96	26.26
US females	25.88	0.10	25.69	26.08
US males	26.91	0.08	26.75	27.08
Native (all)	25.02	0.09	24.85	25.19
Native females	24.39	0.15	24.09	24.69
Native males	25.13	0.11	24.91	25.36
NZ (all)	24.64	0.19	24.27	25.00
NZ females	24.70	0.21	24.27	25.13
NZ males	24.91	0.19	24.53	25.28
**TARSUS LENGTH**				
US (all)	28.17	0.07	28.03	28.30
US Females	27.81	0.08	27.64	27.97
US Males	27.91	0.07	27.78	28.05
Native (all)	29.01	0.08	28.86	29.16
Native females	29.66	0.17	29.32	30.00
Native males	30.10	0.13	29.85	30.35
NZ females	28.15	0.22	27.70	28.60
NZ males	28.67	0.19	28.28	29.05
**DISTAL BEAK LENGTH**				
US (all)	18.43	0.07	18.30	18.57
US Females	17.84	0.08	17.67	18.00
US Males	18.55	0.07	18.41	18.69
Native (all)	17.68	0.07	17.54	17.83
Native females	17.39	0.13	17.14	17.63
Native males	17.70	0.09	17.52	17.88
NZ (all)	16.61	0.16	16.30	16.92
NZ females	16.32	0.16	16.00	16.65
NZ males	16.61	0.14	16.32	16.89
**PROXIMAL BEAK LENGTH**				
US (all)	7.68	0.06	7.56	7.80
US females	8.05	0.08	7.89	8.21
US males	8.37	0.07	8.23	8.50
Native (all)	7.33	0.07	7.20	7.46
Native females	7.01	0.10	6.80	7.21
Native males	7.43	0.08	7.28	7.59
NZ (all)	7.42	0.14	7.14	7.70
NZ females	7.60	0.12	7.37	7.83
NZ males	7.75	0.10	7.55	7.95
**WING LENGTH**				
US (all)	123.50	0.17	123.16	123.84
US females	121.93	0.19	121.56	122.31
US males	123.91	0.16	123.60	124.23
Native (all)	125.91	0.19	125.53	126.29
Native females	124.10	0.39	123.32	124.87
Native males	126.07	0.29	125.49	126.65
NZ (all)	124.18	0.41	123.38	124.99
NZ females	122.89	0.58	121.73	124.05
NZ males	124.60	0.50	123.59	125.60

#### Change over time

##### Fixed effect regression model

Results from fixed effect regressions which controlled for body size and included sex as an interaction term (measurement) ~ (PC1) + (time)*(location)*(sex) showed the following trends (Table 5):

**Table 5:** Results from fixed effect regressions using all starling measurements, PC1 as a fixed effect to control for body size, and time*sex*location as interaction terms.

All Data − fixed effect regressions − (measurement) ~ (PC1) + (TIME)*(LOCATION)*(SEX)								
Source	Increase/Decrease over time	Value	Standard error	DF	t	Pr >|t|	Lower bound (95%)	Upper bound (95%)
**BEAK LENGTH**								
Intercept		24.65	0.16	1190.70	156.94	< 0.0001	24.34	24.95
PC1		0.48	0.06	1193.00	7.57	< 0.0001	0.35	0.60
Native females	Not significant	0.00	0.00	1193.00	− 1.92	0.055	− 0.01	0.00
NZ females	Increase	0.02	0.00	1193.00	4.87	< 0.0001	0.01	0.02
US females	Increase	0.01	0.00	1193.00	7.58	< 0.0001	0.01	0.01
Native males	Not significant	0.00	0.00	1193.00	1.41	0.16	0.00	0.01
NZ Males	Increase	0.02	0.00	1193.00	5.50	< 0.0001	0.01	0.02
US males	Increase	0.01	0.00	1193.00	13.40	< 0.0001	0.01	0.01
**TARSUS LENGTH**								
Intercept		28.93	0.12	1191.20	233.67	< 0.0001	28.68	29.17
PC1		0.96	0.05	1193.00	19.30	< 0.0001	0.86	1.05
Native females	Increase	0.00	0.00	1193.00	2.16	0.03	0.00	0.01
NZ females	Increase	0.03	0.00	1193.00	10.13	< 0.0001	0.02	0.03
US females	Decrease	− 0.01	0.00	1193.00	− 7.45	< 0.0001	− 0.01	0.00
Native males	Increase	0.01	0.00	1193.00	4.39	< 0.0001	0.00	0.01
NZ males	Increase	0.03	0.00	1193.00	11.84	< 0.0001	0.02	0.03
US males	Decrease	− 0.01	0.00	1193.00	− 9.39	< 0.0001	− 0.01	− 0.01
**DISTAL BEAK LENGTH**								
Intercept		17.24	0.09	1190.60	190.90	< 0.0001	17.07	17.42
PC1		1.44	0.04	1193.00	39.93	< 0.0001	1.37	1.52
Native females	Decrease	0.00	0.00	1193.00	− 3.04	0.002	− 0.01	0.00
NZ females	Increase	0.04	0.00	1193.00	21.94	< 0.0001	0.04	0.04
US females	Increase	0.00	0.00	1193.00	7.35	< 0.0001	0.00	0.01
Native males	Decrease	0.00	0.00	1193.00	− 3.44	0.001	− 0.01	0.00
NZ males	Increase	0.04	0.00	1193.00	23.50	< 0.0001	0.04	0.04
US males	Increase	0.01	0.00	1193.00	10.83	< 0.0001	0.00	0.01
**PROXIMAL BEAK LENGTH**								
Intercept		7.40	0.10	1190.90	73.28	< 0.0001	7.20	7.59
PC1		− 0.96	0.04	1193.00	− 23.81	< 0.0001	− 1.04	− 0.88
Native females	Not significant	0.00	0.00	1193.00	− 0.24	0.81	0.00	0.00
NZ females	Decrease	− 0.03	0.00	1193.00	− 14.47	< 0.0001	− 0.03	− 0.03
US females	Increase	0.00	0.00	1193.00	5.28	< 0.0001	0.00	0.00
Native males	Increase	0.01	0.00	1193.00	5.30	< 0.0001	0.00	0.01
NZ males	Decrease	− 0.03	0.00	1193.00	− 14.31	< 0.0001	− 0.03	− 0.02
US males	Increase	0.01	0.00	1193.00	11.21	< 0.0001	0.01	0.01
**WING LENGTH**								
Intercept		122.70	0.29	1188.30	417.24	< 0.0001	122.12	123.27
PC1		2.68	0.12	1193.00	22.76	< 0.0001	2.45	2.91
Native females	Not significant	0.00	0.00	1193.00	0.38	0.70	− 0.01	0.01
NZ females	Increase	0.09	0.01	1193.00	14.67	< 0.0001	0.08	0.10
US females	Not significant	0.00	0.00	1193.00	− 1.16	0.25	− 0.01	0.00
Native males	Increase	0.02	0.00	1193.00	4.91	< 0.0001	0.01	0.02
NZ males	Increase	0.09	0.01	1193.00	17.18	< 0.0001	0.08	0.10
US males	Increase	0.00	0.00	1193.00	2.28	0.02	0.00	0.01

**US:** whole beak length (Females, Males), distal beak length (F, M) and proximal beak length (F, M) increased over time. Tarsus length (F, M) decreased over time, and change in wing length over time increased in males but was not statistically significant in females.

**NATIVE RANGE:** whole beak length was not statistically significant in females or males, and therefore not characterized by change over time. Tarsus length increased over time (F, M). Distal beak length in the native range decreased over time (F, M). Proximal beak length, and wing length increased in males, but was not statistically significant in females.

**NZ:** whole beak length (F, M), tarsus length (F, M), distal beak length (F, M), and wing length increased over time (F, M). Proximal beak (F, M) was the only trait that decreased over time in this population.

##### Linear regressions

Results from linear regressions in the native range show no change in whole beak length over time from all localities over a 206 year-period from 1816 to 2022 (N = 392), (*p value* = 0.426, R^2^ = 0.001) (Fig. 2a), or from a restricted dataset including only England and Germany, from the same time-period (*p value* = 0.620, R^2^ = 0.0007). We do find a statistically significant change over time in the native range in proximal beak length—shorter over time (*p value* =  < 0.0001, R^2^ = 0.042); distal beak length—longer over time (*p value* < 0.0001, R^2^ = 0.056); tarsus length (a proxy for body size)—shorter over time (*p value* < 0.0001, R^2^ = 0.107); and wing length—longer over time (*p value* < 0.0001, R^2^ = 0.0846), (Fig. 2c).

In North American starlings, we find statistically significant changes in all measurements from 1890 to 2020. The change in whole beak length shows the beak getting longer over time (*p value* < 0.0001, R^2^ = 0.076) (Fig. 2a). We also find a statistically significant change in proximal beak length—longer over time (*p value* < 0.0001, R^2^ = 0.2994); while distal beak length is shorter with time (*p value* < 0.0003, R^2^ = 0.0172); tarsus length (a proxy for body size)—shorter over time (*p value* < 0.0001, R^2^ = 0.196); and wing length—shorter over time (*p value* < 0.0001, R^2^ = 0.0206), (Fig. 2c).

In starlings in New Zealand 1927–2017 (N = 68) we find no statistically significant changes over time in any measurement: beak, tarsus, or wing (Fig. 3a–c).

### Comparisons between native range and invasive U.S. population

#### 20 years after introduction to North America

When we compare the native range to North America between 1890 and 1910 we do not find statistically significant differences between means for the whole beak or proximal beak (Table 3, Fig. 4a). Tarsus length (T-test: *p value* < 0.00001) and wing lengths (T-test: *p value* = 0.036) differ between populations, with both measurements smaller in the North American population (Tarsus: NA = 28.53 mm, native = 30.12 mm; Wing: NA = 123.96, native = 125.89 mm), (Table 2, Fig. 4b). Means in Table 2 are based on raw data for museum specimens, compared with fresh specimens which are corrected for comparison with museum specimens.

Beak length averages in the starling population from New Zealand were equivalent to the native range (Beak: NZ = 24.77 mm, native = 24.84 mm, NA = 26.42 mm), but tarsus and wing lengths were closer to that in North America (Tarsus: NZ = 28.50 mm; Wing: NZ = 123.82), (Table 2, Fig. 3d-f).

#### Modern starlings from native range compared with modern starlings in the U.S.

From a random subsample of modern U.S. birds, the average beak length in U.S. was 27.03 mm, this was compared with the average beak length from 137 live-caught birds from a non-agricultural facility in Wales, UK (24.73 mm). The difference in means is 8%.

### Comparison of modern starlings across the United States (East, Central, West; North, South)

Results from ANOVA analyses for eastern, central and western regions of the United States showed no statistically significant differences in group means except for tarsus length values (*p value* = 0.004), with differences between east–west and central-west, where birds from the west have shorter average tarsus lengths. Analyses comparing north and south showed statistically significant differences between tarsus (*p value* = 0.002) and wing measurements only (*p value* =  < 0.00001), where tarsus and wings are shorter in the South versus the North (Table 3, U.S. regions only).

## Discussion

### Body size

North American starlings have shorter tarsi than those in the native range (North America = 27.85 mm, native range = 29.42 mm). Tarsus length can serve as proxy for bird body size^44^. Smaller birds in NA, versus larger birds in the parent population, occurred rapidly on arrival and this trend has persisted in the modern NA starling population today (Figs. 2c,d, 4b). Differences in sex did not explain the variance in tarsus length in NA or NZ and explained only 2% of the variance in this trait for the native range (Table 3). Average tarsus length in New Zealand is 28.5 mm and has increased over time in both sexes. However, only 68 specimens from this population were included, so this trend is preliminary.

**Figure 6:**
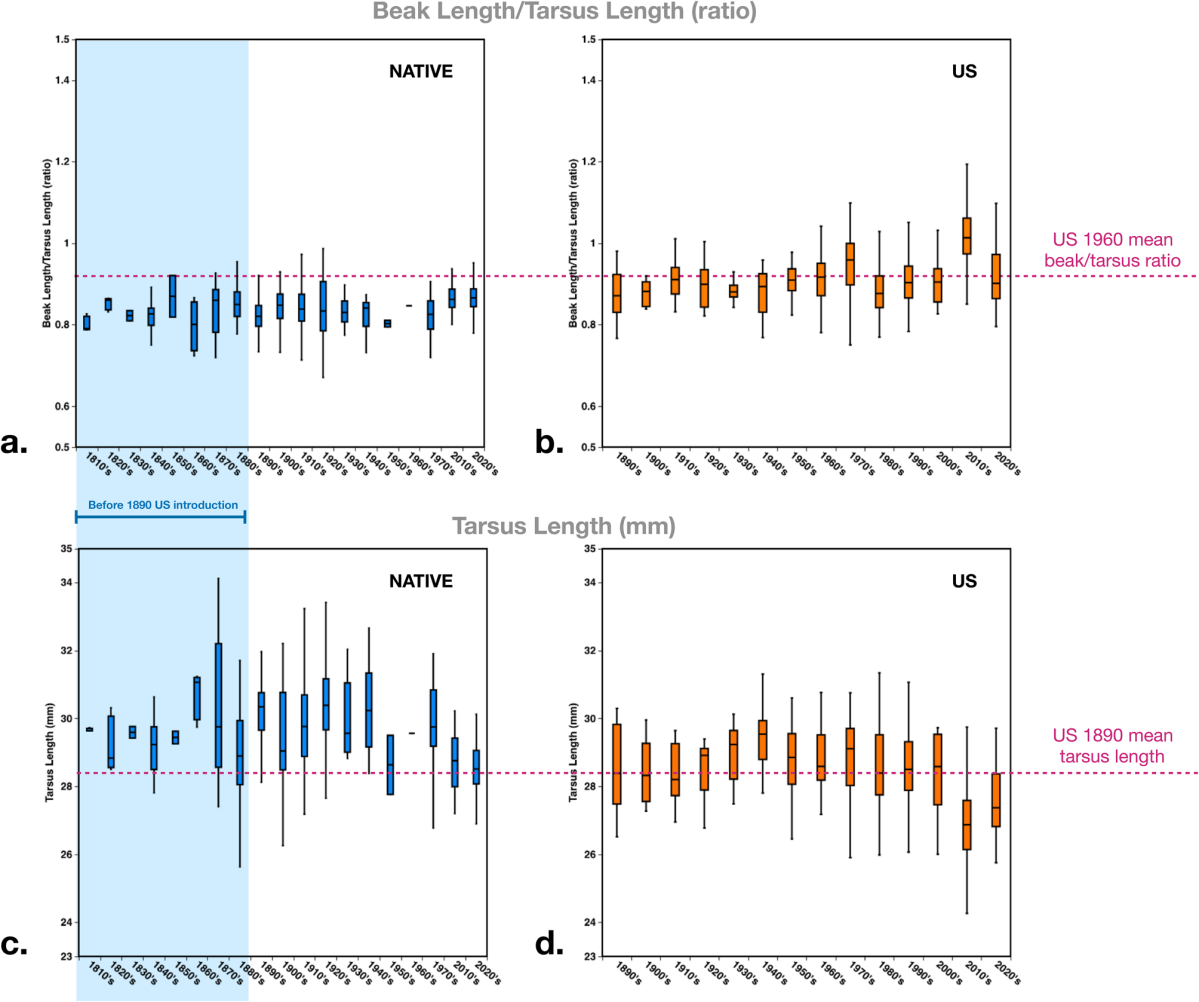
(**a**) Ratio of whole beak length (mm) over tarsus length (mm), to correct for body size, plotted over each decade, native range 1816–2022 (blue), shaded blue area indicates time-period prior to 1890 introduction to the U.S. (**b**) Ratio of beak length/tarsus length in introduced U.S. range (orange) 1890–2020, pink dotted line across indicates mean for U.S. in 1960’s. (**c**) Tarsus length (mm) plotted over each decade, native range 1816–2022 (blue). (**d**) Tarsus length (mm) in introduced U.S. range (orange) 1890–2020, pink dotted line across indicates mean for U.S. in 1890’s.

Reduction in body size has been observed across 52 North American migratory avian taxa and is interpreted as a consequence of global warming^45^. Results here from fixed effect regressions demonstrate that tarsus length in the North American range has decreased over time in both sexes (Table 5). This reduction in body size in NA is evident upon introduction in 1890, which is prior to the 40-year period in Weeks et al. (1978–2016), (see Fig. 6c,d, dotted line). This suggests that global warming is not the primary explanation for this continent-specific trend in North America. Instead, smaller body size in North America could have been selectively advantageous due to warmer average summer temperatures than those in the native range, a pressure which starlings would have experienced during the few first months after introduction.

For the spatial comparison across the United States (2017 only), we find that mean tarsus length decreases further west (averages: East = 26.95 mm, Central = 26.94 mm, West = 26.53 mm), and further south (North = 26.90 mm, South = 26.46 mm), (Table 2; Fig. 5b,c). These subtle trends across the U.S., where tarsus length is smaller in regions further west and south are also supported by ANOVA results which detect a statistically significant difference in this trait between north–south, and east-central-west (Table 3). Differences in temperature, seasonality and/or aridity across the country may play a role in shaping these patterns. Our results here are different to a previous analysis of North American starlings which did not find a relationship between tarsus length variation and any climatic variables^30^. Lack of congruence between this study and ours may be due to differences in sample size, where the previous study included only 168 birds. Therefore, additional analyses with fine-scaled spatiotemporal climatic variables could further clarify the specific drivers of these intriguing patterns.

Taken together, the rapid onset of the change in body size in North America upon arrival, coupled with the results from the spatial analysis, support that smaller body size in North American starlings may be partly due to selection or developmental plasticity in response to warmer summer temperatures than the native range. Though genetic drift upon arrival and/or the founder population of birds randomly consisting of smaller bodied birds cannot be ruled out here.

### Wing length

Consistent with differences in body size, wing length is longer in the native range than in the NA range, and longer in males than females. Fixed effect regressions which control for body size and account for sex show that wing length increased over time in males, but not in females in both the native and North American ranges. This result is consistent with previous studies which have shown that males tend to differentiate more so than females in introduced bird populations^31^. An increase in wing length has also been observed across several North American migratory avian taxa as a compensatory adaptation to maintain migration efficiency when body size declines^45^.

Bitton and Graham found that starlings over a 120-year period in NA showed an increased roundedness of the wing caused by an increase in the length of secondary feathers^4^. They suggest that the wing shape change in NA starlings may have been adaptive, conferring more efficient foraging and predator avoidance.

We also find that wing length is slightly longer in starlings in the Northern U.S. versus those in the South (North = 123.10 mm, South = 121.62 mm), these trends are also supported by ANOVA results (Table 3). This result is also consistent with differences in body size between these two regions, where birds are also slightly smaller in the South than the North.

### Beak length

One of the more robust trends in our data is that whole beak length has increased over time in the invasive North American starling population (in both sexes) with no change over time in the native range. This trend is supported by the results from linear regressions (Fig. 2a,c), and fixed effect regressions which controlled for body size and considered sexes separately (Table 5). ANOVA results show that sex explains 8% of whole beak length variation in the U.S., and 6% in the native range, but this was not a main driver in the overall trends over time. In New Zealand, the average beak length (24.77 mm) was closer to the native range (24.84 mm) than to NA (26.42 mm). Proximal beak length increased over time in the North American range (in both sexes), and in native range males only. Relatedly, sex explained only 1% of proximal beak length in the U.S., and 4% in the native range. Distal beak length increased over time in North America (in both sexes), but decreased over time in the native range (in both sexes), though variation due to differences in beak wear may complicate interpretations of this trend. These changes are evident from a 128-year period in NA, compared with a 206-year period in the native range.

Changes in beak morphology over short time scales have served as a classic example of evolutionary change within bird populations, and between species, for decades^46–48^. These morphological adaptations have been primarily attributed to shifts in food availability, although thermoregulation may also play an important role^49–51^. Additionally, the distal portion of the beak can be subject to different degrees of seasonal wear depending on the environmental substrates with which they regularly interact^52^. Below, we discuss thermoregulation, beak wear, and dietary adaptation as potential mechanisms for the patterns we observe in our beak measurement data.

### Beak thermoregulation

Longer appendages are adaptive as a cooling mechanism in warmer environments (i.e., Allen’s Rule) which has been documented in wild bird populations and tested in controlled experiments^53–55^. Beak development responds to temperature, initiating a plastic response that generates longer beaks in warmer temperatures. Longer beaks then have increased surface area to allow for more efficient cooling in warmer climates^56,57^. A recent review summarizes the evidence that thermoregulation due to climate warming may be driving increased beak length across several avian taxa^51^. Following this trend, beak surface area in introduced Australian starlings is larger in those populations living in hotter and wetter climates^58^.

If global warming were driving the beak length changes we observe in starlings, this trend should be evident across all global populations. Instead, we observe beak length stasis in the native range over 206 years and elongation in North America. This indicates that global warming may not be the primary factor that explains the change observed in NA. Especially since the northern regions of the native range have experienced more extreme changes in average annual temperature over time than equivalent changes in North America^59^. The timing of the observed change in beak length in the U.S. also does not coincide with the most consistent climbing trend in average yearly global temperatures, which began approximately in 1980^60,61^. Instead, we observe an initial overlap in beak length in native and NA starlings after their arrival in North America (1890), but by 1960, lengths in NA are above those found in any specimen from the native range (Figs. 2a, 6a,b).

An alternate climate-driven explanation could be that changes in beak length were impacted due to North American starlings entering a novel climate with more dramatic seasonal temperature fluctuations and higher maximum temperatures than their native range. A recent study of starling beak morphology and genetics in Australia found that single nucleotide polymorphisms (SNPs) correlated with beak surface area were most strongly associated with patterns of daily temperature variation across their range^29^.

We address the possibility of temperature variation as a driver of starling beak morphology in NA by partitioning our modern dataset across the United States within the well-sampled one-year winter period in 2017. We do not find any geographic differences in average beak length across the United States in either east–west or north–south directions (Tables 2 and 3). This suggests that North American starling beak length changes may not be solely the result of thermoregulation due to different regional climates.

Although we found no evidence for differences in beak length across the United States, four individuals from Arizona (collected in 2017 from feedlots) had exceptionally long beaks ranging from 36.83 to 41.34 mm (Image S1). These outliers were removed from all analyses. To determine if this trait was stable in the population from this region, 22 additional individuals were collected from Arizona in January 2020 (from a landfill). None of the additional birds from Arizona showed unusually long beaks, with an average beak length of 25.20 mm (Table 2). See supplementary materials for further discussion of this trend.

### Beak wear

Are starling beaks longer in North America than in their native range because they are experiencing less wear due to differences in environmental substrates or foraging behaviors?

The rhamphotheca, or keratinized outer layer of the beak, grows continuously throughout a bird’s lifetime—and can experience different degrees of wear from seasonal variation in foraging strategies and frequency of behaviors such as bill wiping and preening^52,62–64^. Bill wiping, a mechanism by which birds wear down their beaks by repeatedly drawing them over a surface, has been shown to decrease in starlings feeding on drier foods as compared to foods with a stickier consistency, such as fruit^65,66^. Additionally, in fall and winter, starling beaks are darker colored due to the increased presence of melanin granules which results in a mechanistically harder beak that wears down at a slower rate than their yellow beaks in spring and summer^67^.

Starling’s feeding strategy during warmer months is frequent open bill probing, where they insert their beak into soil, engage the masseter muscle, and rapidly grasp for invertebrates^68^. In the absence of open bill probing, where starlings eat above-ground dry foods from farms primarily in the winter months, beaks may not experience the same degree of abrasion. This seasonal change in foraging behavior, together with harder melanic beaks and a potential decrease in bill-wiping behavior from feeding on drier foods, could collectively contribute to an increased overall beak length in starlings in winter months. The longest beaks we observed in our data were found in the modern USDA dataset, where birds were collected from dairies and feedlots in the winter months, January–March 2016, 2017 (average = 27.33 mm) (Table 2). This suggests a plastic seasonal response to fluctuations in environmental substrate and food availability and not necessarily a developmental or genetic change.

However, our measure of the proximal beak length, from the base of the cranium to the nares, serves as a means of addressing the question of whether seasonal beak wear at the distal end of the beak is driving the differences in beak length we observe. The proximal region of the beak, or frontonasal region, cannot be subject to different degrees of wear throughout the lifetime of the individual; therefore, we use this measurement as a closer approximation of changes at the developmental and genetic levels^69^. We find that the mean proximal beak is 1.166 mm longer (95% CI [1.170,1.163]) in starlings from North America compared with those from the native range and shows a marked increase in length over time (Tables 2 and 5). This supports a potential role for heritable change in beak length over time in North American starlings.

### Dietary adaptation

Beak morphology is known to be partly heritable, and several genes associated with modifications in beak length (COL4A5, BMP4, CaM), beak size (HMGA2), and overall shape (ALX1) show evidence of selection in different avian taxa^70–73^. In light of these elegant studies—which link beak phenotype with genotype—we can better understand how beak morphology evolves in response to natural selection even in the absence of genetic data. Focusing on proximal beak changes in NA starlings, we observe a robust signal where this portion of the beak is getting longer over time. This cannot be explained by lack of beak wear, as stated prior.

Therefore, after considering multiple possible pressures on beak length, we propose that the trend of beak lengthening in North American starlings suggests that dietary adaptation may be contributing to this change. The most dramatic difference between starling diet in the U.S. and their native range is the intensity of their foraging at dairies and feedlots in the U.S., where they consume substantial amounts of food intended for livestock^1^. In this context, grain-based feed consisting of various combinations of grain (e.g., corn, wheat, sorghum), silage, hay, and high energy fat nuggets is distributed in feed troughs or bunks. Since 1960, corn production in the U.S. has increased exponentially, which has also enabled a concurrent expansion of the cattle industry^74^. By the 1960’s feedlot operators in several states were reporting major starling disturbance^75–78^. In our data, 1960 is when we observe a marked increase in proximal starling beak length in the U.S. beyond what is observed in the native range at any time.

Today, the majority of cattle in the U.S. are fed outdoors in winter months, compared with Europe, where cattle primarily graze on grass outdoors in warmer months and in winter are often provisioned indoors (8–10 months in Northern Europe)^79,80^. Starlings in Britain do consume grain feed for livestock, however, the geographic range of this dietary behavior is not equivalent in scale to that in the U.S.—and does not uniformly occur across the entirety of their native range, such as in North Africa or Pakistan^81–84^. In New Zealand, substantially fewer cows are supplemented with corn than in the U.S., and starling damage on farms is concentrated on fruit crops^85,86^.

Starling flocks on U.S. dairies can exceed 10,000 birds and cause an estimated $800 million dollars of annual lost revenue across the country^12,87^. It has been estimated that starlings spend 90 days at livestock facilities in winter, obtain half (0.5) of their winter diet from these facilities, and each bird consumes 0.0625 lbs (0.283 kg) of feed per day^88^. Using the lower bound of current starling population size estimates in the U.S. (85.9 million)^3^, and assuming 0.75% of that population feed at livestock facilities (64.4 million), we estimate that starlings may consume 136,125,000 lbs (61,745 metric tons) of livestock feed per year in the United States. An individual bird can eat up to 2.2 lbs (1 kg) of feed per month, and 1000 birds can consume 630 lbs (286 kg) every hour spent foraging at feedlots^89,90^. Starling feeding experiments show that starlings avoid fibrous food sources such as hay and straw while selecting the more nutritional components of corn and other grains^13^. In these experimental studies, birds preferentially selected steam-flaked corn, a small (5.7 mm) lightweight flat flake, high in starch and distributed to livestock scattered throughout bundles of alfalfa hay^87^. Notably, beak length can be a limiting factor in their access to this vertically distributed food source, as certain feeding tray depths were too deep for their access to grains in these experiments (S. Deliberto, personal communication, 2018). When probing for grass grubs, starling beaks reach less than 2 cm depth into soil, and it has been suggested that longer beaks may also improve their access to invertebrates^58,91^.

A study of the morphology, genetics, and behavior of great tits showed that populations that are provisioned from bird feeders in the UK have evolved longer beaks than continental European populations which are not exposed to bird feeders^70^. Here, we infer that large-scale dairies and feedlots across the United States may have driven starlings to evolve longer beaks to more efficiently forage in this highly modified, energy-rich agricultural landscape. A confounding variable is that starlings regularly utilizing feedlots may also experience less beak wear than those probing for invertebrates in the ground, so careful analyses of seasonal phenotypic changes in all aspects of beak morphology require further examination. The data we report here provide a powerful and unique opportunity to better understand an invasive species’ response to a historically recent, continent-wide anthropomorphic pressure and further illuminate the evolutionary and ecological dynamics between diet, phenotype, plasticity, and adaptation in birds.

## Conclusions

Results presented here show morphological differences between invasive North American starlings and those from their native range, with additional directional change in the North American population through time. In invasive starlings in North America, beak length has increased over 130 years, and tarsus length has decreased. In our sample of modern starlings only, beak length differences are not spatially structured across the United States, consistent with expectations for a heritable trait under stabilizing selection in a panmictic population. This suite of morphological traits in the invasive North American range now contrasts with the starling population in the native range, where beak length has stayed constant during the past 206-years and tarsus length has increased slightly.

European starlings present a rich opportunity to better understand how evolutionary forces such as founder effect, selection, and phenotypic plasticity may have enabled repeated invasions on multiple continents. Humans and invasive European starlings have a deeply entangled ecological history, with several deliberate introductions on multiple continents followed by starlings’ swift and continued exploitation of human modified environments such as urban centers and agroecosystems. Disentangling the precise contributions of evolutionary, ecological, and environmental variables which have shaped these changes present intriguing directions for additional studies.

### Supplementary Information

Supplementary Information.

## Data Availability

The datasets generated during the current study are available from the corresponding author on reasonable request.
