# Supplementary material for: Recent beak evolution in North American starlings after invasion
Source: Sci Rep. 2024 Jan 2;14:140. doi: 10.1038/s41598-023-49623-y (PMC10761893; doi:10.1038/s41598-023-49623-y)
Supplement: Supplementary file 1 — Supplementary Information. [file 41598_2023_49623_MOESM1_ESM.docx]

**SUPPLEMENTAL MATERIAL**

**RECENT BEAK EVOLUTION IN NORTH AMERICAN STARLINGS AFTER INVASION**

Julia M. Zichello^1,2^, Shelagh T. DeLiberto^3^, Paul Holmes^4^, Agnieszka A. Pierwola^5^, Scott J. Werner^3^

**ARIZONA OUTLIERS:**

We found no evidence for differences in beak length across the United States with the exception of four individuals from Arizona. Four birds collected in 2017 had exceptionally long beaks ranging from 36.83-41.34 mm (Image S1). These outliers were removed from all analyses. To determine if this trait was stable in the population from this region, 22 additional individuals were collected from Arizona in January 2020. None of the additional birds from Arizona showed unusually long beaks, with an average beak length of 25.20 mm (Table 2). Although the four initial outliers may have been a random occurrence and not indicative of a larger pattern, it is worth noting the congruence between this finding and those of the most recent large-scale genomic study of starlings in the US. Hofmeister et al. (2021) did not find any evidence for genetic structuring of starling populations across North America—with the exception of a slightly elevated F_ST_ value differentiating populations from Arizona and New Mexico. Stable isotope data also supports that starlings from this region exhibit lower migration rates than in other parts of the country (Werner et al. 2020). A lower degree of genetic admixture between starling populations in and around Arizona could potentially act to maintain genetically distinct populations, which could give rise to unique beak morphologies such as those observed here. Continued monitoring of starling populations across the country with finer-scale geographic sampling may further clarify the transience or stability of this morphological pattern.


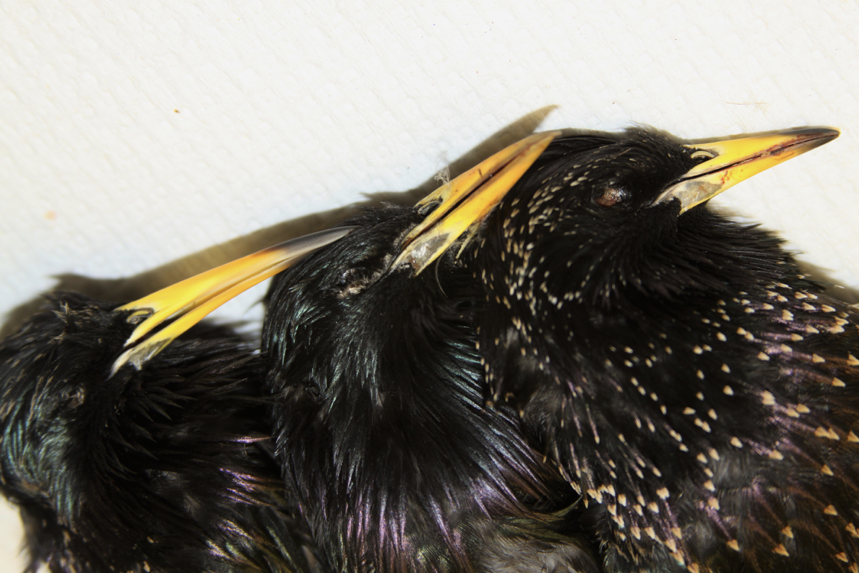


Image S1
